# Laparoscopic cholecystectomy performed by a surgical care practitioner: a review of outcomes

**DOI:** 10.1308/rcsann.2023.0058

**Published:** 2024-04-25

**Authors:** S Odogwu, S Morris, S Addison, S Abbott

**Affiliations:** ^1^ Walsall Healthcare NHS Trust, UK; ^2^ University Hospitals Birmingham NHS Foundation Trust, UK

**Keywords:** Nurse practitioner, Cholecystectomy, laparoscopic, Clinical nurse specialist, Development, staff

## Abstract

**Introduction:**

Surgical care practitioners (SCPs) are non-medical workers involved in various aspects of the management of surgical patients. The role includes assisting and performing surgical procedures. More than 60,000 laparoscopic cholecystectomies (LC) are performed annually in the UK. With ever-increasing pressure on waiting lists, it is important to look at fully utilising the skills of our entire workforce. We report what we believe is the first published series of LC performed by an SCP.

**Methods:**

A retrospective review of a prospectively collected database was performed. The primary outcome was any complication requiring intervention. Secondary outcomes were minor complications, operative time, length of stay, conversion and readmission.

**Results:**

In total, 170 patients were operated on. Indications were biliary colic in 127 (74.7%), cholecystitis in 30 (17.6%) and pancreatitis in 13 (7.6%). Mean operating time was 65min (range 35–152min). Fifty-three operations were assisted by a consultant, 110 by a specialist or associate specialist grade (SAS) doctor and 7 by a core trainee (CT2). Some 139 (81.7%) patients were discharged on the day of surgery and 24 (14.1%) stayed one night in hospital. There were no major complications. Five patients required readmission, three with pain and two with port site infections. There were no conversions or transfusions required.

**Conclusions:**

There is a paucity of published data on surgical outcomes of procedures performed by SCPs. With a structured, supervised approach, SCPs could be trained to take on more complex procedures and further strengthen the surgical workforce. This study demonstrates that elective LC can be safely performed by an appropriately trained and supervised SCP.

## Introduction

There has been a need in recent years to review the composition of the surgical workforce in the National Health Service. The reduction in trainees’ working hours and a chronic shortage of medical staff have resulted in the development of several advanced roles for non-medical practitioners. One of these roles is the surgical care practitioner (SCP).^1^

The SCP role was initially introduced in the 1990s. To enhance the surgical workforce and provide additional flexibility in the face of the reduction in junior doctors’ hours, the role was formalised, and a training framework was introduced in 2006.^2^ The surgical workforce is changing, and an expanding work force of non-medical practitioners is contributing significantly to patient care. There is a wide variation in scopes of practice across this group of professionals.^3^

In recent years there has been an increasing emphasis on establishing more robust and well-defined training programmes. The Royal College of Surgeons of England (RCS England) recognises that current models of healthcare delivery need to change. There is evidence that the surgical care team can positively impact on service delivery, training and patient experience. A recent review by the RCS England notes that “Extended practitioner roles complement, but do not replace, surgeons or medical staff. They enhance the capability of the surgical team and should evolve together within the team. Their educational development should not compromise the training of future surgeons.”^4^ Due care should be given to ensure that the training of extended roles does not come at the expense of surgical trainees’ access to training opportunities.

Our review of the literature has revealed that there are few data on the types of operations and outcomes of surgery performed by SCPs.^5^ The first reports of SCPs performing parts of operations were from cardiothoracic surgery. Other reports have covered minor surgery such as lipomas and sebaceous cysts and varicose vein surgery.^6,7^ These studies concluded that non-medical practitioners were effective and had good surgical outcomes with these procedures. An orthopaedic study comparing failure rates in hip aspirations looked at outcomes of an SCP undertaking the procedure regularly and compared it with registrars and consultants. The SCP had a statistically significant lower rate of failed aspirations compared with the other two groups.^8^

The SCP in our unit (SM) qualified as a registered general nurse in 1994. Once qualified she began her career as a scrub nurse and later a theatre sister, working across the general surgical specialties. In 2004, as part of the Department of Health's ‘New ways of working’ initiative, mentioned previously, she was given the opportunity to embark on the SCP course through the University of Greenwich. She qualified as a colorectal SCP in 2006. Over the following 10 years she was mentored by the surgical team, developing her skills as an assistant as well as learning surgical operative skills. As her operative skills developed, she became competent in minor surgery progressing to umbilical hernia repair and inguinal hernia repair. Because she had also spent a lot of her time as an SCP assisting with major laparoscopic colorectal procedures, her laparoscopic skills had become quite advanced. This was when it was decided that she should develop her skills further and learn to perform laparoscopic cholecystectomies (LC). The supervising consultants (SO, SAd, SAb) all agreed that the SCP had the necessary understanding of pathophysiology and anatomy. She also had the necessary experience in terms of having seen and assisted several hundred cases and performed parts of many of these procedures. Her surgical ability was also judged to be at the necessary level by all three trainers. She had been consistently performing the procedure at level 4 under direct observation with the consultant assisting.

A programme was put in place with a plan to progress from consultant-assisted procedures (with the consultant scrubbed) through specialist or associate specialist (SAS) doctor-assisted procedures (SAS doctor scrubbed with the consultant immediately available if needed) and finally to independent practice with a consultant available for attendance in theatre if required.

## Methods

We performed a retrospective review of a prospectively collected database. All operations performed by SM from June 2015 to November 2019 were reviewed. Patient demographics, operative details, readmission rates and complication rates were reviewed.

The primary outcome measure was any complication requiring intervention (Clavien–Dindo grade 3); that is, intra-abdominal collections, bile leak, bile duct injury or retained stones.

Secondary outcomes included, operative time, conversion, length of stay, readmission and minor complications (Clavien–Dindo grades 1 and 2).

### Ethical approval

Informed consent regarding the surgery and operating surgeon was obtained from all patients. Ethical approval for the study was sought from the local ethics committee and not deemed necessary because this was classed as service improvement and service evaluation.

### Statistical analysis

Qualitative variables are expressed as number of cases and percentages, whereas the quantitative variables are expressed as mean, range and standard deviation (SD). To compare the qualitative variables, the chi-squared test or the Fisher's exact test were used. The two sample *t-*test and the chi-squared test were used for the quantitative variables. The statistical analysis was performed with IBM® SPSS® Statistics version 22.

## Results

One hundred and seventy patients were included in the series. Demographics were as documented in Table 1. The average age was 47 years and the majority were American Society of Anesthetists (ASA) graded I and II.

**Table 1:** Patient demographics

Male (*n*)	26
Female (*n*)	149
Age (years)	47.41 ± 16.469 (SD) [range 16–83]
BMI (kg/m^2^)	32 ± 6.253 (SD)
ASA1 (*n*)	59
ASA2 (*n*)	81
ASA3 (*n*)	30
Indication for surgery (*n*)	
Biliary colic	127
Cholecystitis	30
Pancreatitis	13

ASA = American Society of Anesthesiologists; BMI = body mass index

Indications for surgery were biliary colic in 127 (74.7%), cholecystitis 30 (17.6%) and pancreatitis in 13 (7.6%).

Fifty-three operations were assisted by a consultant, 110 by a senior SAS grade doctor and 7 by a core trainee (CT2). Mean operating time overall was 64.34min (± 21.24 SD). The median was 59min (range 21–152min). The operating time, length of stay and readmission rates in relation to whether the case was assisted by a consultant, SAS doctor or core trainee are summarised in Table 2.

**Table 2:** Operative data

Assistant	*n*	Mean operative time (min)	Length of stay (days)				No. of readmissions	No. of transfusions
			0	1	2	2+		
Consultant	53	63.62 ± 27.89	47 (88.6)	3 (5.6)	3 (5.6)	0	2 (3.7)	0
SAS	110	65.95 ± 27.84	87 (79.1)	19 (17.3)	3 (2.7)	1 (0.9)	3 (2.7)	0
CT2	7	68.71 ± 24.83	5 (71)	2 (29)	0 (0)	0 (0)	0 (0)	0
Total	170	64.34 ± 27.72	139 (81.7)	24 (14.1)	6 (3.5)	1 (0.6)	5 (2.9)	0

Values in parentheses are percentages. Operative time is given as mean ± SD

CT2 = core trainee; SAS = specialist or associate specialist doctor

There were no major complications requiring intervention.

There was no statistically significant difference between operating times when the SCP was assisted by a consultant compared with when she was assisted by an SAS or CT grade doctor (*p* = 0.594, two-tailed *t*-test).

There was, however, a statistically significant difference between day case and overnight admission rates when the SCP was assisted by a consultant compared with when she was assisted by an SAS or CT grade doctor. Operations assisted by a consultant had a day case rate of 88.6% compared with 79.1% for those assisted by a middle grade doctor (*p* = 0.017, chi-squared test).

There was also no significant difference between readmission rates (*p* = 0.53, chi-squared test).

There were four readmissions. Two patients presented with postoperative pain. Both had computed tomography scans that showed no abnormalities and both were discharged with no treatment other than analgesia. There were two superficial wound infections. Both were treated with antibiotics alone. One of the patients with a wound infection had undergone a simultaneous umbilical hernia repair at the time of LC.

There were no conversions and no patients required blood transfusion.

## Discussion

The training of the SCP to perform LC began as an ad hoc process in which she would carry out parts of the procedure. Initially inserting ports, then dissecting the gall bladder off the liver bed, later progressing to dissection of Calot’s triangle and clipping and division of the cystic artery and duct after obtaining a critical view. It quickly became apparent that she had the aptitude to perform the procedure and consequently the supervising consultants put in place a programme to enable her to develop her skills further.

This involved initially performing the procedures assisted either by a consultant or one of two senior SAS doctors both of whom perform independent LC. At this stage, the supervisor would take over if there was difficulty in progressing.

Once the SCP was consistently performing the procedure satisfactorily under direct supervision and guidance, we progressed to her performing LC independently. Satisfactory performance was defined as meeting the criteria for level 4 as described in the Intercollegiate Surgical Curriculum Programme (ISCP) for General Surgery (2016).^9^ Procedures were performed with the consultant assisting, but not providing guidance, or with a consultant observing unscrubbed. Subsequently, procedures were performed with assistants of varying degrees of experience and the consultant not in theatre, but immediately available for advice or help if required.

It could be argued that the amount of consultant input was high and potentially not cost-effective. The level of input could have been less, but because this was a novel approach we felt that it would be safer to err on the side of caution. In the longer term and with the introduction of a formal, centralised training programme, the process could be streamlined and made more efficient.

Our results have shown the feasibility of SCPs performing LC. Patient selection was limited to those from the pooled general surgery list. This generally excludes patients who are expected to be particularly difficult, such as those with previous multiple attacks of severe cholecystitis, abscess, localised perforation, radiological drainage or cholecystostomy. Patients with morbid obesity (BMI > 40) and previous laparotomy were excluded.

The results show operating times and complications in keeping with published series.^10^ Our day case rates were excellent in comparison with published data.^11^ There were no major complications requiring surgical, radiological or endoscopic intervention.

Loss of the old firm structure and reduction in junior doctors’ hours has resulted in a situation whereby there is little or no continuity of staffing. This is exacerbated by frequent rotation of juniors. This can result in consultants rarely having the same assistant, with potentially negative effects on teamworking, efficiency and, in extreme cases, safety.^12,13^ Development of the alternative workforce gives reliability and continuity. Having a regular assistant familiar with the surgeon, team, operation and equipment reduces variation, stress and potentially reduces risk. The SCP can provide a degree of constancy in a team. Certainly, in our team the consultants find doing a difficult case assisted by the SCP easier and less stressful than operating with a comparatively inexperienced trainee.

The SCP is in a unique position of having a good understanding of the reality of the doctor's role as well as the roles of nurses and operating department practitioners. They are in a unique position to bridge this gap. Experienced SCPs can also help to train, supervise and support both the theatre team and junior doctors.^14^

There has been a perception that SCPs may impact on the trainee surgeon’s educational opportunities, particularly in theatre. This has been raised in a consensus recommendation by the Association of Surgeons in Training (ASIT).^15^ However, a survey in the same document also revealed that 72% of ASIT trainees surveyed felt that SCPs improved service delivery and 58% felt that they improved patient care. This training issue needs to be managed carefully and can be addressed with appropriate rota planning and communication. In our 18-year experience of having an SCP, she has always been happy to step back when a trainee wishes to operate. She is, as far as possible, assigned to lists in a way that does not impact negatively on training. She also provides a skilled, experienced assistant for the more senior trainees and can provide teaching for the more junior trainees. This experience has also been reported in other units.^16^

Kingsnorth reported that the process of training an SCP to perform inguinal hernia was “very long … and not cost-effective”.^17^ This was not our experience, because our SCP was a quick learner with excellent judgement and surgical skills. Learning is multifactorial, and skills vary. Further work to improve ways of identifying individuals with the necessary attributes are needed.^18,19^ The training of SCPs has been addressed jointly by the Royal Colleges of Surgeons of England and Edinburgh and a structured Curriculum Framework for Surgical Care Practitioners has been developed.^20^ The aim is to “set the clinical, technical and professional standards” for the SCP workforce of the future. The intention is that this will result in standardisation of training and competencies.

Acceptance of the non-medical workforce by patients is crucial to the success of the SCP. There is currently little research looking at patient perception of SCPs. In our series, informed consent was taken by the SCP. Patients were aware that she would be operating under the supervision of the consultant. None of the patients in our series raised any concerns. Good-quality patient surveys may help to improve our understanding of patient perceptions of SCPs. A clear understanding of the roles, scope of practice and limitations of the SCP is essential.

## Conclusions

We have reviewed the outcomes of LC performed by an SCP. Our results show that the procedure can be performed safely and effectively with very low complication rates and high day case rates. Fully utilising the skills and abilities of all members of the extended surgical team will be integral to the effective delivery of healthcare in the future.

## Acknowledgements

The authors wish to thank Liz Askew, Information and Knowledge Specialist at Walsall Healthcare NHS Trust for her help with the literature search for this manuscript.
